# The Role of High-Resolution Lung Computed Tomography to Distinguish Between Fibrosing Hypersensitivity Pneumonitis and Usual Interstitial Pneumonia

**DOI:** 10.3390/life15121867

**Published:** 2025-12-05

**Authors:** Dmitry A. Kuleshov, Svetlana Yu. Chikina, Galina V. Nekludova, Igor E. Tyurin, Sergey N. Avdeev

**Affiliations:** 1 Federal Central Research Institute of Tuberculosis, Moscow 107564, Russia; dimson1994@mail.ru; 2 Pulmonology Department, I.M. Sechenov First Moscow State Medical University (Sechenov University), Healthcare Ministry of Russia, Moscow 119991, Russia; nekludova_gala@mail.ru (G.V.N.); avdeev_s_n@staff.sechenov.ru (S.N.A.); 3 Pulmonology Scientific Research Institute, Federal Medical and Biological Agency of Russian Federation, Moscow 115682, Russia; 4 Russian Federal Academy of Continued Medical Education, Healthcare Ministry of Russia, Moscow 123995, Russia; igortyurin@gmail.com

**Keywords:** hypersensitivity pneumonitis, interstitial lung disease, usual interstitial pneumonia, idiopathic pulmonary fibrosis, centrilobular nodules, craniocaudal distribution, honeycombing

## Abstract

**Background**: Hypersensitivity pneumonitis (HP) is an interstitial lung disease (ILD) caused by repeated exposure to inhaled antigens in susceptible subjects. High-resolution computed tomography (HRCT) of the lungs is the leading diagnostic method for ILDs, but in some cases HRCT findings are not sufficient to distinguish HP and other ILDs, particularly, fibrotic HP (fHP) and usual interstitial pneumonia (UIP). **Objective**: The aim of this study was to develop HRCT criteria to diagnose fHP in patients with a UIP-like pattern. **Methods**: In this retrospective study, we analyzed HRCT scans of patients with fHP and a UIP-like pattern who underwent lung biopsy, and patients with idiopathic pulmonary fibrosis (IPF) and a UIP pattern in HRCT. **Results**: We included 51 patients with confirmed fHP and 24 patients with IPF/UIP in the analysis. IPF/UIP patients were older, were prevalently males, and did not have any systemic autoimmune diseases or risk factors for other ILDs. fHP patients were younger, with an equal number of males and females, and were more likely to be exposed to environmental antigens. HRCT abnormalities in the fHP group predominated in the lower lung areas or were diffuse in axial scans, whereas IPF/UIP patients mostly demonstrated a diffuse craniocaudal distribution and subpleural axial predominance. Centrilobular nodules and mosaic attenuation were present significantly more often in the fHP group; honeycombing, traction bronchiectasis, and emphysema prevailed in IPF/UIP patients. In the logistic regression analysis, patients with fHP and IPF/UIP differed in the presence of centrilobular nodules, honeycombing, and in both craniocaudal and axial distributions of HRCT abnormalities. In the ROC analysis, the combination of centrilobular nodules, honeycombing, and diffuse axial and craniocaudal distributions can predict the diagnosis of fHP (AUC, 0.953 ± 0.022; 95%CI, 0.910–0.995; *p* < 0.001). Mosaic attenuation and reticulation did not change the probability of fHP. **Conclusions**: The most significant HRCT features of fHP compared to the UIP pattern were centrilobular nodules, honeycombing, and a diffuse axial and craniocaudal distribution of abnormal findings. Reticulation, mosaic attenuation, and GGO do not increase the probability of fHP.

## 1. Introduction

Hypersensitivity pneumonitis (HP) is an interstitial lung disease (ILD) caused by repeated exposure to inhaled antigens in susceptible subjects. The disease is characterized by the immune-mediated inflammatory response in the lower respiratory tract, mainly in the terminal bronchioli and alveoli, and in the pulmonary interstitium [1,2].

High-resolution computed tomography (HRCT) of the lungs is the leading diagnostic method for ILDs and allows for the description of pulmonary abnormalities in detail, differentiating between a range of inflammatory and other interstitial lung diseases, evaluating a clinical course, and monitoring the disease under a treatment. Nevertheless, in some cases, HRCT findings are not sufficient to distinguish HP and other ILDs: such cases typically require lung tissue biopsy [3]. Fibrotic HP (fHP) and usual interstitial pneumonia (UIP) are the most difficult to distinguish.

According to an official ATS/JRS/ALAT clinical practice guideline, fHP is characterized by features of lung fibrosis, such as irregular linear opacities or coarse reticulation with lung distortion, traction bronchiectasis, and honeycombing, and can also include a UIP pattern with basal and subpleural distribution of honeycombing with or without traction bronchiectasis [4]. Extensive ground glass opacity (GGO) could also present with superimposed subtle features of lung fibrosis. The distribution of fibrosis may be random in both axial and craniocaudal planar reconstructions, or can predominate in the mid-lung areas with relatively spared lower lung areas. Variant distributions of lung fibrosis can be peribronchovascular and subpleural in axial images, with the predominance in the upper lung areas. HRCT abnormalities indicative of small airway disease should also be found, such as ill-defined centrilobular nodules, GGOs with mosaic attenuation, or a three-density pattern [4].

UIP is common in patients with idiopathic pulmonary fibrosis (IPF), but can be found in other fibrosing ILDs including fHP. A UIP pattern includes honeycombing with or without traction bronchiectasis, irregular thickening of interlobular septa, and reticulation, superimposed with mild GGO and with subpleural and basal predominance [5,6]. Therefore, both patterns, fHP and UIP, have similar HRCT features: this significantly complicates making a confident diagnosis. T.Tateishi reported the accuracy of radiologic diagnosis of fHP as 78 to 80% [7]. So, some cases require histopathologic examination to distinguish these diseases. This is an important issue given the different therapeutic approaches to fHP and IPF [6]. However, lung biopsy is not available in some cases, including severe respiratory failure, significant pulmonary hypertension, comorbidities, etc.

The aim of this study was to develop HRCT criteria to differentiate fHR with a UIP-like pattern from IPF/UIP.

The study was approved by the Ethics Committee of Sechenov University (Moscow, Russia) (approval number: 06-16; approval date: 12 April 2016).

## 2. Materials and Methods

*Data collection.* In this retrospective study, we used a database of patients admitted to a university respiratory clinic from 2016 to 2021. We included patients with fHP and a UIP-like pattern who underwent a lung biopsy to confirm the diagnosis, and patients with idiopathic pulmonary fibrosis (IPF) and a UIP pattern in HRCT. Diagnosis of IPF was made according to an official ATS/ERS/JRS/ALAT clinical practice guideline, 2018 [5], based on the clinical probability of IPF (age > 60 years, smoking history, no systemic autoimmune diseases, and no risk factors for other ILDs). According to the current guidelines, typical or probable UIP pattern on lung HRCT scans in a patient with clinical probability of IPF and no clinical concern for an alternative diagnosis does not need histopathological confirmation [6], so IPF patients with HRCT patterns of typical or probable UIP did not undergo a lung biopsy. fHP patients had a history of environmental exposure and a clinical course compatible with HP [4]. They underwent a video-assisted thoracoscopic (VATS) lung tissue biopsy because clinical findings were suggestive for HP, but HRCT findings were in line with a UIP-like pattern.

*HRCT analysis.* HRCT scans of patients were analyzed by two independent radiologists that were experienced in the diagnosis of ILDs and were blinded to the final diagnosis. Interobserver discrepancies were resolved by consensus. The HRCT scans were performed with a slice thickness of ≤1.5 mm in the supine position at the end of inspiration. Reconstruction algorithms, such as multiplanar reconstruction (MPR), maximal intensity projection (MIP), and minimal intensity projection (MinIP), were used. HRCT scans were analyzed using Slicer 3D 4.11.20210226 software and the Chest Imaging Platform integrated software package v.4.10 (NA-MIC on the basis of Brigham and Women’s Hospital, Boston, MA, USA).

We analyzed the presence and the distribution of the following HRCT signs: GGO, consolidation, centrilobular nodules, mosaic attenuation, emphysema, reticulation, honeycombing, and traction bronchiectasis. All the signs were defined according to the glossary of terms for thoracic imaging from the Fleischner Society [8].

*Statistical analysis.* The findings were statistically analyzed using SPSS Statistics software, version 23.0 (IBM, Armonk, NY, USA), and R Software, version 4.0.2. Data were expressed as median (Me) and interquartile range (IQR) or as a percentage. Comparisons between groups were made using Pearson’s χ2 test or Fisher’s two-sided exact test (for classified data) or Mann–Whitney’s U-test (for continuous data) A *p* value of <0.05 was considered statistically significant. Spearman’s correlation coefficient with subsequent logistic regression model analysis was used to analyze the correlation between variables. The receiver operating characteristic (ROC) curves were used to analyze the diagnostic value of the regression models.

## 3. Results

### 3.1. Patients

**Figure 1:**
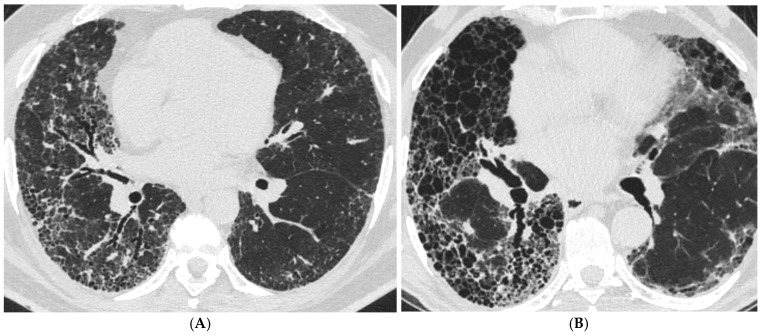
Axial CT scans of fHP with UIP-like HRCT pattern (**A**) and IPF/UIP (**B**). Extensive reticulation, traction bronchiectasis, air-filled cysts (probably honeycombing), and mild GGO are seen in both scans.

Fifty-seven patients with fHP confirmed by lung biopsy and a UIP-like HRCT pattern and twenty-four patients with IPF/UIP were retrospectively selected from the database of the University Respiratory Clinic, Sechenov University, Moscow, from 2016 to 2021. We excluded 6 patients with fHP due to insufficient biopsy specimens. Therefore, 75 patients were included in the analysis: 51 patients with confirmed fHP with a UIP-like HRCT pattern and 24 patients with IPF/UIP. CT-scans of a patient with fHP and a UIP-like HRCT pattern (A) and a patient with IPF/UIP (B) are given in Figure 1.

Basic characteristics of the patients are given in Table 1. IPF/UIP patients were >60 y.o., were predominantly males, and did not have any systemic autoimmune diseases or risk factors for other ILDs. fHP patients were younger, with an equal number of males and females, and were more likely to be exposed to environmental antigens. They had fibrotic lung HRCTs resembling a UIP pattern. Smoking history did not differ significantly between the groups, but numerically patients in the IPF/UIP group were more severe smokers. In the fHP group, the causative exposure was known in 19 (33.3%) patients.

### 3.2. HRCT Patterns in the Groups

Centrilobular nodules and mosaic attenuation were seen significantly more often in the fHP group, whereas honeycombing, traction bronchiectasis, and emphysema prevailed in the IPF/UIP patients. The frequencies of GGO, reticulation, and consolidation did not differ significantly between the groups (Table 2).

GGOs were superimposed to traction bronchiectasis in a majority of cases in both of the groups; no differences in the predominant distribution of these changes were seen between the groups.

**Figure 2:**
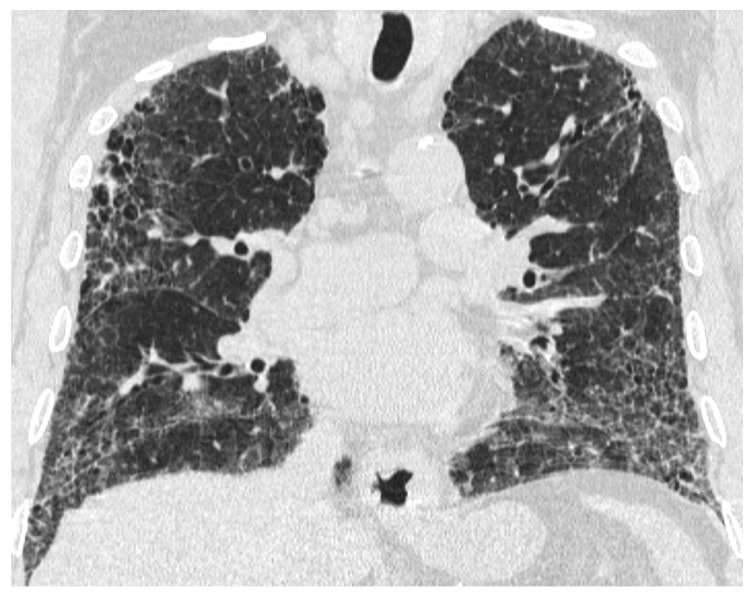
An axial CT scan of a patient with IPF/UIP. HRCT abnormalities are mostly diffuse without craniocaudal predominance.

HRCT abnormalities in the fHP group predominated in the lower lung areas or were diffuse in axial scans, whereas IPF/UIP patients mostly demonstrated diffuse craniocaudal distribution (Figure 2) and a subpleural predominance in axial distribution. Upper lung predominance and peribronchovascular distribution were rare in fHP and were not found in IPF/UIP patients (Table 3).

### 3.3. Logistic Regression Analysis

The patients with fHP and IPF/UIP differed in the presence or absence of centrilobular nodules, honeycombing, and in both craniocaudal and axial distributions of abnormal HRCT findings (Table 4).

### 3.4. ROC-Analysis

**Figure 3:**
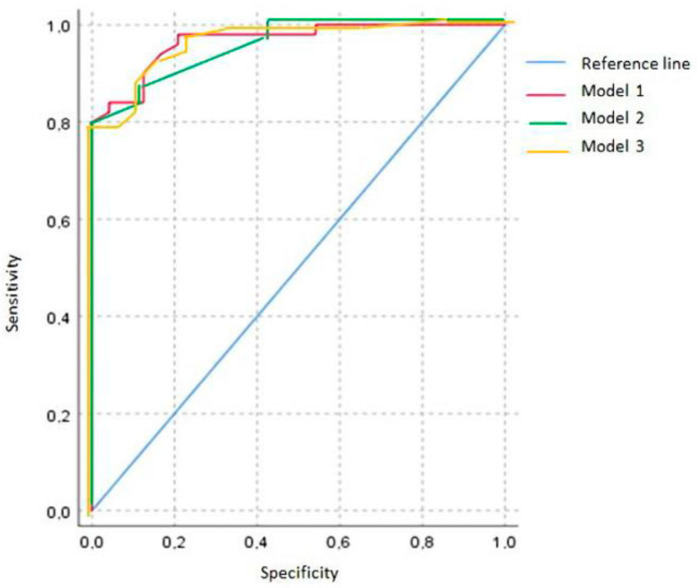
ROC analysis of different fHP diagnostic models.

We included HRCT findings that were significantly related to the diagnosis of fHP in the logistic regression model (centrilobular nodules, honeycombing, and diffuse axial and craniocaudal distributions). ROC analysis revealed that the combination of these HRCT findings can predict the diagnosis of fHP (the area under the curve (AUC) was 0.953 ± 0.022 (95%CI, 0.910–0.995; *p* < 0.001)) (Figure 3, model 1). When mosaic attenuation and reticulation were added to the model, the AUC was not significantly changed (0.965 ± 0.019; 95%CI, 0.928–1.002; *p* < 0.001, and 0.967 ± 0.018; 95%CI, 0.932–1.001; *p* < 0.001, respectively) (Figure 3, models 2 and 3, respectively).

## 4. Discussion

In this retrospective study, we demonstrated that the combined use of HRCT findings (centrilobular nodules, honeycombing, and diffuse axial and craniocaudal distributions) was excellent for predicting the diagnosis of fHP (AUC 0.95; *p* < 0.001). Mosaic attenuation and traction bronchiectasis did not affect the probability of radiological diagnosis of fHP.

A HRCT pattern of fHP can mimic a UIP pattern, as signs of pulmonary fibrosis, primarily honeycombing and traction bronchiectasis, can be seen in both the diseases along with GGO and reticulation. HRCT features of small airway disease, including centrilobular nodules and mosaic attenuation, are considered as typical for fHP and are not common in a UIP pattern [4]. However, in the progressive course of the disease, the extent of centrilobular nodules and GGOs decreases [9]. In a study by Marinescu et al., typical fHP patterns corresponded to an fHP clinical diagnosis in 65% of patients, and typical and probable UIP patterns corresponded to a diagnosis of IPF in 66% and 57% of patients, respectively [10].

Traction bronchiectasis is an important feature of pulmonary fibrosis and is an obligatory sign of a UIP pattern [6]. We found traction bronchiectasis in 100% of IPF/UIP patients and in two thirds of patients with fHP. We suppose that this sign did not become an independent diagnostic marker of fHP in our study due to its high prevalence in both of the groups. On contrary, in the study by Sumikawa et al. [11], traction bronchiectasis together with GGO, peribronchovascular opacities in the upper lung, and random distribution were significant features for fHP diagnosis in multivariate analysis compared to IPF. However, the AUC of the fHP diagnostic model in the study by Sumikawa et al. was 0.733 (95% confidence interval [CI], 0.655–0.811, *p* < 0.001) in the test group and 0.630 (95% CI, 0.504–0.755, *p* < 0.047) in the validation group, compared to 0.953 in our study.

Honeycombing is also an important HRCT feature of pulmonary fibrosis and can be seen in any fibrosing ILD, particularly one with a UIP pattern. In our study, honeycombing was found in 100% of patients with IPF and in 23.5% of patients with fHP. The presence of honeycombing decreased the probability of having fHP by 46% compared to IPF.

GGO has been considered for a long time as an inflammatory, primarily exudative, lesion of pulmonary parenchyma. Currently GGO is well recognized as a possible consequence of pulmonary fibrosis if combined with other fibrotic findings [12,13]. In pulmonary fibrosis, GGO results from a compaction of the lung tissue and a reduction in lung volume due to fibrosis. However, GGO is not a reliable sign for the diagnosis of fHP as GGO could also be found in a UIP pattern [7,14]. In the study by Tateishi et al., extensive GGO was found in 37% of patients with IPF/UIP [7]. The authors suggested that extensive GGO should be excluded from the suggestive findings of fHP due to its low specificity and low positive predictive value. Chelala et al. reported GGO in 44% of IPF patients and in 74% of HP patients, without a significant difference [15]. We also did not find a significant difference in the number of fHP and IPF/UIP patients with GGO.

Mosaic attenuation is an independent predictor of non-fibrosing HP, but in fHP areas of mosaic attenuation could be replaced by fibrosis [9]. Moreover, mosaic attenuation is also an important sign in fHP. The progressive extension of areas of mosaic attenuation increased the probability of fHP [16]. In our study, mosaic attenuation did not reliably predict fHP diagnosis.

Another important point is the distribution of HRCT abnormalities. In a typical UIP pattern, the pathological features predominate in subpleural and basal parts of the lungs [6] compared to a typical fHP pattern with upper and mid-lung predominance [4]. In our study, about 80% of IPF patients demonstrated diffuse craniocaudal distribution and 25% had diffuse axial distribution. According to guidelines, this is not common for a UIP pattern [6]. However, the upper lobe predominance in a UIP pattern is described in several publications. T.Tateishi et al. reported no significant difference in the distribution of radiological abnormalities between fHP and IPF/UIP. They found the upper or mid-lung predominance in 7% of IPF/UIP patients and in 20% of fHP patients. The extensions of abnormal findings in the upper, mid-lung, and lower lung areas in IPF/UIP patients were 40.0, 38.9, and 57.1%, respectively [7]. L.Chelala et al. in a retrospective study described random craniocaudal and axial distributions of HRCT abnormalities in 29% and 21% of IPF patients, respectively [15]. We suppose that such results could be explained by a greater extension of pulmonary fibrosis in patients with advanced IPF when fibrosis involves the upper lung areas; in this case, the difference in the extent of abnormal findings in the upper and lower lung areas disappears. On the contrary, abnormal HRCT findings predominated in the lower lung areas in 34% to 42% of fHP patients and were diffuse both in craniocaudal and axial distributions in 48% to 63% of patients [13,15,17]. Therefore, the lower lung predominance of HRCT abnormalities in fHP is not rare in clinical practice.

HRCT fibrotic abnormalities are often seen in COVID-19 survivors. In the majority of patients interstitial lung alterations improved over time, with a significant reduction at 90 days after recovery [18]; however, sometimes post-COVID interstitial lung abnormalities can persist for a longer time [19,20]. This is a challenging situation for physicians because post-COVID fibrotic abnormalities include GGO, reticulation, and, in some patients, honeycombing. This could make it significantly more difficult to determine if ILD is purely post-COVID sequelae or if it is a chronic fibrosing ILD that had not been diagnosed before. Moreover, according to recent publications, post-COVID HRCT abnormalities could be bilateral with lower lobe (up to 73%) and peripheral (up to 68%) predominance, with the development of honeycombing in 21% of cases [21]. Bronchiectasis, fibrosis, and honeycombing are also described in COVID patients [22]. Differentiation between post-COVID ILD and other ILDs is crucial because post-COVID ILD is known as a non-progressive disease and, therefore, does not need antifibrotic treatment [23]. On contrary, most other ILDs can progress, with a risk of respiratory failure and poor prognosis. Further investigations are needed to define diagnostic markers allowing differentiation of these diseases.

Our study has several limitations. Firstly, this was a retrospective and a single-center study that is associated with a certain risk of biases. Secondly, the sample size was relatively small and the difference in the patient groups was quite large. This is due to the fact that IPF is a less prevalent ILD compared to fHP. The small sample size, particularly in the IPF/UIP group, should be noted as a factor that can make logistic regression models unstable. To overcome this limitation, we analyzed statistical data using tests for small samples. Thirdly, we were not able to analyze end-expiratory HRCT scans that could help to reveal air trapping, a key feature of HP. This could be an explanation as to why mosaic attenuation was not a diagnostic feature to differentiate between fHP and IPF/UIP in our study.

In conclusion, the most significant HRCT features of fHP compared to a UIP pattern were centrilobular nodules, honeycombing, and diffuse axial and craniocaudal distributions of abnormal findings. Reticulation, mosaic attenuation, and GGO do not increase the probability of fHP.

## Figures and Tables

**Table 1:** Basic characteristics of patients.

Parameter	fHP (*n* = 51)	IPF/UIP (*n* = 24)	*p*
Age, years (Me, IQR)	55 (42–60)	67 (63, 5–73)	<0.001
Gender (M/F), *n*	27/24	22/2	<0.001
Smoking history, pack-years	20 (13–32)	30 (25–45)	0.241
BMI, kg/m^2^	28.0 (24.9–31.6)	29 (25.5–31.4)	0.192

Note. fHP, fibrosing hypersensitivity pneumonitis; IPF, idiopathic pulmonary fibrosis; UIP, usual interstitial pneumonia.

**Table 2:** Frequency of different HRCT findings in fHP and IPF/UIP patients.

HRCT Findings, *n* (%)	fHP, *n* = 51	IPF/UIP, *n* = 24	*p*
Centrilobular nodules	24 (47.1%)	1/24 (4.2%)	0.003
Mosaic attenuation	27 (52.9%)	5 (20.8%)	0.009
Emphysema	22 (43.1%)	16 (66.7%)	0.048
GGO	46 (90.2%)	24 (100%)	0.168
Consolidation	6 (11.8%)	1 (4.2%)	0.421
Reticulation	50 (98.0%)	24 (100%)	1.000
Honeycombing	12 (23.5%)	24 (100%)	0.000
Traction bronchiectasis	34 (66.6%)	24 (100.0%)	0.001

Note. fHP, fibrosing hypersensitivity pneumonitis; IPF, idiopathic pulmonary fibrosis; UIP, usual interstitial pneumonia.

**Table 3:** Spatial distribution of abnormal HRCT findings.

Types of Spatial Distribution		fHP, *n* = 51	IPF/UIP, *n* = 24	*p*
Craniocaudal distribution, *n* (%)	upper lung areas	4 (7.8%)	0 (0.0%)	0.299
	lower lung areas	22 (43.1%)	4 (16.7%)	<0.001
	diffuse	25 (49.0%)	20 (83.3%)	0.006
Axial distribution, *n* (%)	peribronchovascular	2 (3.9%)	0 (0.0%)	1.000
	subpleural	15 (29.4%)	18 (75.0%)	<0.001
	diffuse	34 (66.7%)	6 (25.0%)	<0.001

**Table 4:** Diagnostic values of main HRCT signs of fHP vs. UIP pattern.

HRCT Findings	OR	95% CI	*p*
Centrilobular nodules	74.62	2.96–187.95	0.009
Mosaic attenuation	1.536	0.081–2.171	0.170
Honeycombing	0.54	0.33–0.89	0.005
Traction bronchiectasis	0.25	−0.44–0.57	0.804
Diffuse craniocaudal distribution	0.15	0.03–0.86	0.033
Diffuse axial distribution	12.77	2.34–69.81	0.003

Note. OR, odds ratio; CI, confidence interval.

## Data Availability

The original contributions presented in this study are included in the article. Further inquiries can be directed to the corresponding author.

## Abbreviations

The following abbreviations are used in this manuscript:

HP	Hypersensitivity pneumonitis
HRCT	High-resolution computed tomography
fHP	Fibrotic hypersensitivity pneumonitis
UIP	Usual interstitial pneumonia
IPF	Idiopathic pulmonary fibrosis
ILD	Interstitial lung disease
AUC	Area under curve
ATS	American Thorascic Society
JRS	Japanese Respiratory Society
ALAT	Asociacion Latinoamericana de Torax
IQR	Interquartile range
MPR	Multiplanar reconstruction
MIP	Maximal intensity projection
MinIP	Minimal intensity projection
GGO	Ground glass opacity
